# Escalating trends and burden of HIV among men who have sex with men in Bangladesh: A newly emerging concentrated epidemic

**DOI:** 10.1016/j.bsheal.2026.07.003

**Published:** 2026-07-09

**Authors:** Hemayet Hossain, Md. Hasan Ali, Md. Abdur Nur Sakib, Md. Mahfujur Rahman

**Affiliations:** a Department of Anatomy & Histology, Sylhet Agricultural University, Sylhet 3100, Bangladesh; b Department of Veterinary Science and Animal Husbandry, Teesta University, Rangpur 5404, Bangladesh; c Faculty of Veterinary, Animal and Biomedical Sciences, Sylhet Agricultural University, Sylhet 3100, Bangladesh; d Department of Medicine, Sylhet Agricultural University, Sylhet 3100, Bangladesh

**Keywords:** Men who have sex with men (MSM), Human immunodeficiency virus (HIV), New epicenter, Bangladesh

## Abstract

Bangladesh has historically maintained a low-level human immunodeficiency virus (HIV) epidemic; however, recent surveillance indicates a shifting burden toward men who have sex with men (MSM). This study aimed to assess temporal trends and comparative HIV risk among key populations in Bangladesh. National HIV surveillance data from 2016 to 2025 were analyzed using univariate logistic and Poisson regression models. MSM showed a markedly higher risk (odds ratio [OR] = 5.89; 95% confidence interval [CI]: 5.05–6.90; *P* < 0.001) of HIV infection compared with people who inject drugs and a significantly higher incidence rate (log-incidence rate ratio [IRR] = 1.2, 95% CI: 1.1–1.3; *P* < 0.001). Temporal analysis revealed a steep increase in HIV risk among MSM, with odds rising from 2.20 in 2020 to 15.2 in 2025 relative to 2019. These findings indicate an emerging concentrated HIV epidemic among MSM in Bangladesh, underscoring the need for strengthened, context-specific prevention strategies, particularly considering known socio-structural barriers.

## Highlights

• Men who have sex with men (MSM) showed a significantly higher risk of human immunodeficiency virus (HIV) than people who inject drugs (PWID) (odds ratio [OR]  = 5.89; *P* < 0.001).
• HIV incidence among MSM was higher than PWID (log-incidence rate ratio [IRR] = 1.2, 95% CI: 1.1–1.3; *P* < 0.001).
• Odds of HIV infection among MSM increased sharply from 2020 to 2025 (OR up to 15.2).
• Findings indicate a rapidly emerging concentrated HIV epidemic among MSM in Bangladesh.

## 1 Introduction

Historically, Bangladesh has maintained a low human immunodeficiency virus (HIV) prevalence (<0.1%) and is classified as a low-level HIV epidemic. National HIV prevention and control efforts have primarily focused on selected key populations with a higher burden of infection, particularly people who inject drugs (PWID) [1]. Nevertheless, an increase in HIV incidence rates has been shown through the results of integrated biological and behavioral surveillance (IBBS) surveys focused on men who have sex with men (MSM) over the years. The prevalence among MSM in Dhaka, Bangladesh increased from <1% in 2016 to >3% by 2021 [2]. There have been some serious changes noticed within the Bangladesh scenario regarding HIV. According to recent statistics from the National AIDS/STD Programme (NASP), 1,891 new HIV infections were reported between November 2024 and October 2025, exceeding the 1,438 new infections reported in 2024 and representing the highest annual number documented in Bangladesh to date [3], [4]. Cultural, legal, and religious reasons contribute to the stigma and marginalization of MSM, thereby limiting access to both prevention and care services [5]. Historically, early prevention strategies and surveillance systems may have played a significant part in controlling the spread of HIV in the MSM community [6]. However, recent national surveillance reports demonstrate an alarming epidemiologic transition, with an increase in HIV diagnosis rates among MSM.

In Bangladesh’s social and cultural context, this trend is of particular significance. Same-sex sexual behavior remains both illegal and socially unacceptable, and persistent legal, cultural, and religious stigma discourages men who have sex with men (MSM) from disclosing their sexual identity [7]. As a result, MSM are frequently compelled to conceal their identities, face severe discrimination related to their sexuality, and experience substantial barriers to accessing HIV prevention, testing, and care services [8]. Such environments allow for a delay in diagnosing the disease and for continued transmission of the virus among MSM. To better understand recent trends in epidemiology and provide information for developing specific intervention approaches, we have analyzed the national surveillance data to look at how HIV is spreading among MSM compared to other groups of individuals within Bangladesh.

## 2 Methods

This study was conducted with the national HIV surveillance data published by the Directorate General of Health Services from National AIDS/STD Control (https://asp.portal.gov.bd/). For identification of a new key population group, we analyzed last 10 years HIV surveillance data from 2016 to 2025. The 8 key population groups were PWID – people who inject drugs; FSW – female sex workers; MSM – men who have sex with men; MSW – male sex workers; TG – transgender people; Migrant – migrant workers/mobile populations, FDMN – forcibly displaced Myanmar nationals, and the Other KPs – key populations that are vulnerable to HIV infection, including populations at higher risk due to behavioral, social, or structural factors not categorized above. All data were analyzed at the population level.

Univariate logistic regression models were used to estimate odds ratios (ORs) for HIV infection across key population groups. Poisson regression models were also fitted to estimate incidence rate ratios (IRRs), adjusting for year and testing volume using the total number tested as an offset. Temporal trends among MSM were assessed using year-wise logistic regression with 2019 as the reference year.

## 3 Results and discussion

Across key populations, MSM highlighted one of the highest HIV burdens. A univariate analysis revealed that MSM demonstrated a significantly higher level of HIV infection compared to the PWID group (OR = 5.89, 95% CI: 5.05–6.90; *P* < 0.001). Men who have sex with men (MSM) exhibited a significantly higher HIV incidence rate than PWID (log[IRR] = 1.2, 95% CI: 1.1–1.3; *P* < 0.001), corresponding to an incidence rate more than three times higher relative to the PWID reference group (Table 1). MSW (log[IRR] = 0.35), migrant workers (log[IRR] = 0.61), FDMN (log[IRR] = 0.33), and other key populations (log[IRR] = 1.1) also revealed significantly higher HIV incidence rates, whereas FSW and TG populations showed a significantly negative association of HIV incidence compared with PWID, with log[IRRs] of approximately −2.3 and −2.0, respectively.

**Table 1:** Univariate logistic and Poisson regression analyses of HIV risk across key population groups in Bangladesh.

HIV key population groups	Univariate logistic regression			Poisson regression		
	OR	95% CI	*P*-value	Log (IRR)	95% CI	*P*-value
PWID	Ref.	–	–	Ref.	–	–
FSW	0.18	0.12, 0.25	<0.001	−2.30	−2.60, −2.00	<0.001
MSM	5.89	5.05, 6.90	<0.001	1.20	1.10, 1.30	<0.001
MSW	2.37	2.00, 2.82	<0.001	0.35	0.23, 0.47	<0.001
TG	0.18	0.12, 0.25	<0.001	−2.00	−2.30, −1.80	<0.001
Migrant	2.39	2.02, 2.85	<0.001	0.61	0.50, 0.72	<0.001
FDMN	1.90	1.59, 2.28	<0.001	0.33	0.21, 0.45	<0.001
Other KPs	4.11	3.51, 4.84	<0.001	1.10	1.00, 1.20	<0.001

Abbreviations: CI, confidence interval, OR, odds ratio, IRR, incidence rate ratio; Ref., reference; PWID, people who inject drugs; FSW, female sex workers; MSM, men who have sex with men; MSW, male sex workers; TG, transgender people; Migrant, migrant workers/mobile populations (individuals who move across regions or borders for work or livelihood); FDMN, forcibly displaced Myanmar nationals (Rohingya population residing in Bangladesh); Other KPs, other key populations vulnerable to HIV infection, including populations at higher risk due to behavioral, social, or structural factors not categorized above.

**Fig. 1:**
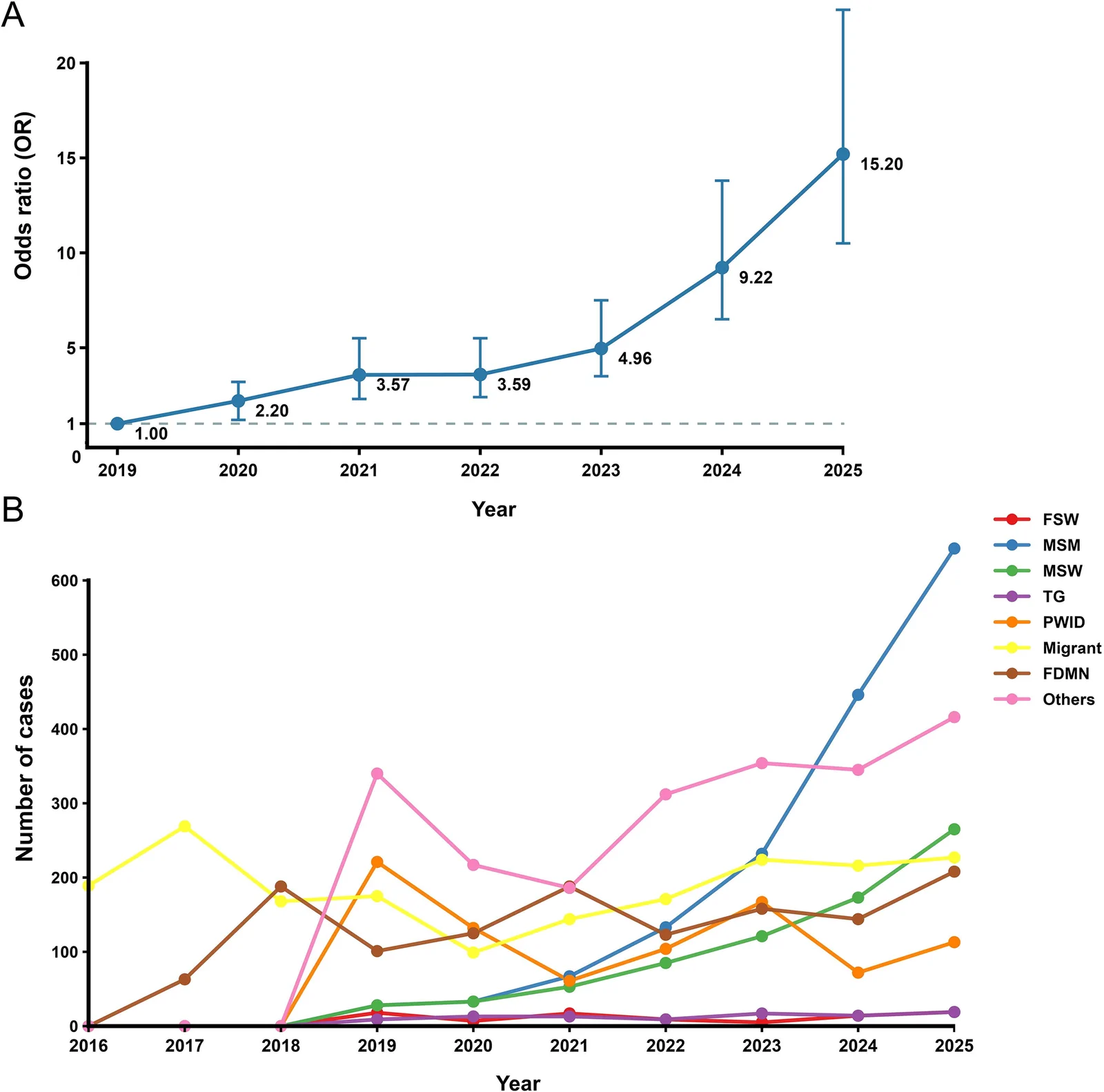
Temporal trends in HIV burden among key populations and changes in the odds of HIV infection among men who have sex with men (MSM) in Bangladesh. A) Year-wise ORs for HIV infection among MSM from 2019 to 2025, estimated using logistic regression. Error bars represent 95% CIs, and the dashed horizontal line at OR = 1 indicates the null hypothesis (no association). Panel A begins in 2019 because MSM cases were first reported in the surveillance dataset from that year, allowing OR estimation. B) Trends in HIV cases among MSM and other key population groups, Bangladesh, 2016–2025. Abbreviations: HIV, human immunodeficiency virus; MSM, men who have sex with men; FDMN, forcibly displaced Myanmar nationals; FSW, female sex workers; MSW, male sex workers; PWID, people who inject drugs; TG, transgender people; OR, odds ratio; CI, confidence interval.

Temporal analysis revealed significantly (*P* < 0.001) pronounced and accelerating increase in HIV among MSM (Fig. 1A; Table 2). Using 2019 as the reference year, the odds of HIV infection among MSM showed a consistent upward trend over time, rising to 2.20 in 2020, 3.57 in 2021, 3.59 in 2022, 4.96 in 2023, 9.22 in 2024, and reaching 15.2 in 2025 (*P* < 0.001). This trend indicates a consistent increase over time, with notable acceleration observed in recent years (Fig. 1).

**Table 2:** Temporal trend of MSM (men who have sex with men) positivity among tested individuals.

Year	Total tested	MSM positive cases	χ^2^	*P*-value
2019	938,069	28	782.87	<0.0001
2020	502,165	33		
2021	628,312	67		
2022	1,241,625	133		
2023	1,568,184	232		
2024	1,621,661	446		
2025	1,421,347	643		

Surveillance data further supported these results. The absolute number of newly diagnosed HIV cases among MSM rose markedly after 2019, paralleling a nationwide increase in HIV diagnoses despite expanded testing coverage. Over time, MSM constituted a growing share of new HIV cases, accounting for nearly one-third of all diagnoses in the most recent surveillance years (Fig. 1B).

Among MSM groups, a chi-square test showed a highly significant association between year and MSM positivity (*χ^2^* = 782.87*, P* < 0.0001). MSM cases increased markedly from 28 in 2019 to 643 in 2025, indicating a clear temporal rise over the study period.

Although increased testing may partially explain higher case detection, several findings suggest that expanded testing alone does not account for the observed trends [5], [9], [10]. First, the magnitude of relative risk among MSM remained consistently high across analytical approaches. Second, year-wise odds increased disproportionately compared with overall testing growth. Third, MSM continued to show elevated risk compared with multiple other key populations, including those traditionally prioritized in prevention programs.

The sociocultural environment likely plays a critical role in shaping these outcomes. Fear of disclosure, discrimination, and legal repercussions discourages many MSM from seeking timely testing or preventive services. Limited availability of MSM-friendly healthcare settings and insufficient community-based outreach further restrict access. Consequently, infections may remain undiagnosed for prolonged periods, facilitating onward transmission within hidden sexual networks.

## 4 Limitations

The analysis relied on passive national HIV surveillance data, which may be affected by underreporting and reporting inconsistencies. The hidden and stigmatized nature of the MSM population in Bangladesh may also have resulted in underestimation of the true HIV burden. In addition, possible misclassification of key population categories and the observational nature of the study may limit causal interpretation of the findings. Despite these limitations, the consistent upward trends highlight an important emerging public health concern.

## 5 Conclusions

HIV among MSM in Bangladesh has shifted from a marginal concern to a rapidly emerging public health challenge. Despite low overall prevalence, MSM now bear a disproportionate and growing burden of HIV infection. Cultural and religious norms, and structural barriers to healthcare continue to jeopardize progress in HIV control. Immediate and targeted interventions are warranted to scale up confidential, rights-based, and culturally appropriate HIV prevention and testing services for MSM. Strengthening community engagement, reducing stigma within healthcare settings, and ensuring early linkage to care are essential to prevent further escalation and potential spillover into the wider population.

## Ethics statement

This study analyzed secondary monitoring data from routine public health surveillance and publicly available sources. No identifiable personal information was collected or used, and no human participants were directly involved. Therefore, institutional ethics committee approval and informed consent were not required.

## Conflict of interest statement

The authors declare that there are no conflicts of interest.

## Author contributions

**Hemayet Hossain:** Writing – review & editing, Writing – original draft, Visualization, Validation, Software, Methodology, Investigation, Data curation, Conceptualization. **Md. Hasan Ali:** Writing – original draft, Methodology, Investigation, Formal analysis, Data curation. **Md. Abdur Nur Sakib:** Writing – original draft, Methodology, Investigation, Formal analysis, Data curation. **Md. Mahfujur Rahman:** Writing – review & editing, Writing – original draft, Visualization, Validation, Supervision, Software, Resources, Methodology, Investigation, Data curation, Conceptualization.

## Declaration of generative AI and AI-assisted technologies in the writing process

During the preparation of this work the author(s) used ChatGPT-4 (OpenAI) in order to assist with English language editing, grammatical correction, sentence refinement, and improvement of manuscript readability and clarity. After using this tool/service, the author(s) reviewed and edited the content as needed and take(s) full responsibility for the content of the published article.
